# Statins as potential therapeutic drug for asthma?

**DOI:** 10.1186/1465-9921-13-108

**Published:** 2012-11-24

**Authors:** Cheng Yuan, Lin Zhou, Jiyun Cheng, Jingying Zhang, Yue Teng, Mao Huang, Ian M Adcock, Peter J Barnes, Xin Yao

**Affiliations:** 1 Department of Respiratory Medicine, The First Affiliated Hospital of Nanjing Medical University, 300 Guangzhou Road, Nanjing, China; 2 Airway Disease Section, National Heart and Lung Institute, Imperial College, Dovehouse Street, London, UK

**Keywords:** Statins, Asthma, Anti-inflammatory, Lung function

## Abstract

**Background:**

Statins are lipid-lowering agents that also exhibit pleiotropic effects in decreasing oxidative stress and inflammation. There have been several published studies reporting the use of statins in the treatment of asthma patients, but their results are not consistent. The aim of this study is to determine whether statins are beneficial for asthma administration, and explore the potential covariables that may affect their clinical effectiveness.

**Methods:**

A systematic literature search was performed in PubMed, Embase and Cochrane Center Register of Controlled Trials from inception to September 2012. Randomized controlled trials (RCT), retrospective studies and controlled clinical trials which reported the use of statins in the treatment of asthma patients were eligible. Quality evaluation was conducted for RCT using Jadad criteria.

**Results:**

A total of 18 articles were included. In our study, we found no conclusive evidence to demonstrate that statins could enhance the lung function in asthmatics, although, they may reduce airway inflammation. Additionally, the results were not consistent across studies with respect to symptoms, quality of life, maintenance medication, asthma hospitalization/emergency department (ED) visits.

**Conclusions:**

Statins may reduce airway inflammation in asthmatics, without having a significant effect on lung function. Further large sample and multicenter clinical trials are needed to confirm this and to see if there are more responsive phenotypes of asthma.

## Introduction

Chronic airway inflammation plays a major role in the pathophysiology of asthma, and is also associated with airway hyperresponsiveness. Glucocorticoids,leukotriene modifiers, and anti-IgE antibody are the main anti-inflammatory medications to keep asthma under clinical control chiefly through their anti-inflammatory effects [1,2]. However, specific subpopulations of individuals including smokers [3], obese asthmatics [4,5] and non-Th2-high asthmatics [6] respond poorly to the above medications.

Statins,inhibitors of hydroxymethylglutaryl coenzyme A (HMG-A) reductase,can inhibit the mevalonate pathway and the synthesis of downstream intermediates including farnesylpyrophosphate (FPP) and geranylgeranylpyrophosphate (GGPP), which post-translationally modify small guanosine triphosphatases (GTPases) [7,8]. GTPases may play a role in the pathophysiology of asthma, because they could enhance airway smooth muscle contraction and proliferation, and increase airway hyperresponsiveness [9,10]. Studies have demonstrated that statins reduce the total inflammatory cell infiltrate and eosinophilia in bronchoalveolar lavage fluid in an animal model of asthma [11] and inhibit the airway smooth muscle proliferation and contraction in vitro [12].

In 2009, a retrospective study by Stanek et al. [13] showed that statin therapy was independently associated with a significant 33% relative risk reduction for recurrent asthma-related hospitalization/emergency department (ED) events. Recently, several studies have been performed in asthmatics to investigate the clinical effectiveness of statins in asthma. Some studies suggested that short-term treatment with statins could increase lung function, enhance the anti-inflammatory effect of inhaled corticosteroids (ICS), and improve the Asthma Control Questionnaire (ACQ) and Asthma Quality of Life Questionnaire (AQLQ) [14-16]. However, other studies failed to replicate these results [17-19]. Therefore, we performed a systematic review to see whether asthmatic patients could benefit clinically from statins, and explore potential factors that may affect their clinical effectiveness.

## Methods

### Search strategies

A systematic literature search was conducted by two investigators (C Y and Y T) independently in Pubmed, Embase and Corchrane Center Register of Controlled Trials from database inception to September 2012, with the reference lists browsed at the same time. The following terms were used for statins: “hydroxymethylglutaryl coenzyme a reductase inhibitors”, “HMG-CoA reductase inhibitors”, “simvastatin”, “lovastatin”, “pravastatin”, “fluvastatin”, “atorvastatin”, “cerivastatin”, “rosuvastatin”, “pitavastatin”, “statin”, “statins” and “compactin”. The following terms were used for asthma:“asthma”, “bronchial spasm”, “bronchoconstriction”, “bronchial hyperreactivity”, “airway inflammation”, “wheeze” and “wheezing”. There was no language restriction.

### Study selection criteria

Studies which selected asthmatics exposed to statins as the trial group and asthmatics unexposed to statins as the control group, or made assessment on the effect of statins by comparing it with the baseline were eligible. Also abstracts without full text that provided information on asthmatics about lung function outcomes, airway inflammation, or the quality of life were included.

Studies that conducted in vitro or in animal models were excluded (Additional file 1).

### Data extracted

According to the search strategy and select criteria, two investigators (C Y and Y T) respectively reviewed the titles, abstract and full articles, we obtained the eligible studies. However, 10 studies, including studies only published as abstracts which did not provide enough information and intervention studies without a placebo control were discussed by the authors and their inclusion resolved by consensus after review by X Y. For each study, relevant data were directly derived from the paper including the demographic data, administration, type of study, duration of treatment, outcomes assessment, conclusions etc.

### Quality evaluation

Quality evaluation was conducted for each randomized controlled trial. Four RCT studies were excluded from the quality evaluation because they were published in the form of an abstract and therefore we were unable to extract enough information from them. Quality evaluation is consistent with the Jadad criteria (scoring was made according to descriptions for randomization, double blinding, withdrawals and dropouts, maximum score 5) [20]. A poor score was defined as less than 2, and a good score was defined as 3–5 (Table 1).

**Table 1:** Results of the quality evaluation for selected RCT studies

**Study**	**randomisation**	**double-blind**	**description dropouts**	**Jadad Scale**
Menzieset al., [21]	1	2	1	4
Hothersallet al., [22]	1	2	1	4
Maneechotesuwan et al., [15]	1	2	1	4
Cowan et al., [25]	1	2	1	4
Braganza et al., [16]	2	2	1	5

Jadad criteria allocate a point each for randomization, double-blind design, and description of dropouts. If randomization and double-blind concealment are assured, an additional 2 points are added. If randomization or double-blind concealment is not assured, a point is deducted for each. A trial with a score of 3 or more is regarded as high quality. Data from trials with scores of 3 or more were grouped and analysed separately from those scoring less than 3.

## Results

**Figure 1:**
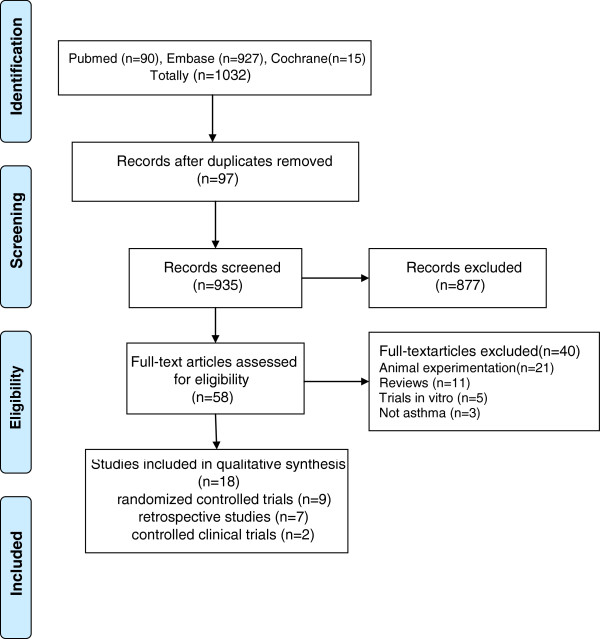
Results of the systematic literature search.

Figure 1 shows the results of the systematic literature search. A total of 1032 articles were reviewed, of which 877 were irrelevant and 97 were duplicate studies, and therefore they were excluded from the study after screening the titles. Of the remaining 58 relevant studies, 21 were concerned with animal experiments, 11 were reviews, 5 were in vitro trials, and 3 were not related to asthma. Finally, 18 articles were included in our study: 9 RCT (4 published as abstracts), 7 retrospective studies (4 published as abstracts), and the remaining two were controlled clinical trials (published as abstracts) comparing statins with the placebo or baseline. There was a significant difference in the methodology, demographic data, baseline characteristics and outcome measures between the retrospective studies and the RCT studies. Additionally, insufficient data were available in the 5 RCT studies whose designs were relatively rigorous by scoring four or more points. We tried to contact the authors by email to obtain the original data without success. A meta-analysis was, therefore, impracticable and systematic qualitative appraisal was performed.

### Lung function

Of the eight RCT studies reporting lung function results in asthma patients, six showed that statins did not improve lung function [16,19,21-24]. One demonstrated that simvastatin was associated with minor improvement in FEV_1_ (p<0.01) in the absence of steroids [25] and another demonstrated that atorvastatin promoted clinical and functional improvement in night-time symptoms, cough, daily symptoms and FEV_1_ in severe asthma patients [14]. Three retrospective studies showed inconsistent results. Pagovich et al. demonstrated that statins were associated with improvement in peak flow (PF) measurements (p<0.0001) [26], whereas Adams et al. suggested that statins did not have a beneficial effect on lung function in asthma patients [27]. Ostroukhova et al. intriguingly reported that FEV_1_ became worse in asthma patients who received statins [18]. Finally, in a small controlled clinical trial with only 9 asthmatic patients, atorvastatin failed to improve lung function [28]. Overall, there was no evidence that statins improved lung function in asthmatic patients (Table 2).

**Table 2:** Studies which report lung function after statin treatment

**Study**	**Study type**	**Group**	**Sample (Trial/Control)**	**Duration of treatment**	**Results (statin group compared with the control group)**
Ostroukhova et al., [18]	Retrospective study	Statin exposed vs statin unexposed	50(24/26)	2 years	3% to 5% median worsening of FEV_1_ ↓
Braganza et al., [16]	RCT	Atorvastatin(40mg/day) vs placebo	71	4weeks	No significant difference
Moskovljevicet al., [28]	Controlled clinical trial	Atorvastatin(10mg/day) vs placebo	9	4 weeks	No significant difference
Menzies et al., [21]	RCT	Simvastatin(20mg/day,40mg/day)vs placebo	16	4 weeks	No significant difference
Hothersall et al., [22]	RCT	Atorvastatin(40mg/day) vs placebo	54	8 weeks	No significant difference
Cowan et al., [25]	RCT	Simvastatin(40mg/day) vs placebo	43	4 weeks	PEF, FEV_1_ (p<0.01) ↑
Pagovich et al., [26]	Retrospective study	Atorvastatin, simvastatin vs baseline	70	4 weeks	PF (p<0.0001) ↑
Foumani et al., [19]	RCT	Atorvastatin(40mg/day) vs placebo	67	8 weeks	No significant difference
Fahimi et al., [23]	RCT	Atorvastatin(10mg/day) vs placebo	17	4 weeks	No significant difference
Feschenko et al., [14]	RCT	Atorvastatin+ICS+Salbutamol vs ICS+salbutamol	31	4 weeks	Morning PEF, FEV_1_ ↑
Adams et al., [27]	Retrospective study	Statin exposed vs statin unexposed	539	Not mentioned	No significant difference
Moini et al., [24]	RCT	Atorvastatin(40mg/day) vs placebo	62	8 weeks	No significant difference

PEF=Peak Expiratory Flow; FEV_1_= Forced expiratory volume in one second; PF= Peak flow.

↑ The lung function was improved in statin group compared with the control group, ↓ an opposite result.

### Airway inflammation

Three randomized double-blind clinical trials demonstrated that statins could decrease induced sputum cell counts in asthmatics, including eosinophils [15,25] and macrophages [22]. Evidence also suggested that simvastatin had an anti-inflammatory activity in reducing serum ECP and CRP levels [29]. Additionally, Menzies et al. demonstrated that simvastatin led to a 0.86 geometric mean fold decrease (95% CI, 0.7 to 1.04; P=0.15) in fractional exhaled nitric oxide (F_E_NO), and a −0.18 doubling dilution shift (95% CI, –1.90 to 1.55; P=1.0) in methacholine hyperresponsiveness [21]. In contrast Braganza et al. [16] showed that the cell counts in induced sputum were similar after atorvastatin and placebo treatment. Overall, statins reduced airway inflammation in asthma patients (Table 3).

**Table 3:** Studies which report airway and serum inflammation after statin treatment

**Study**	**Study type**	**Group**	**Sample (Trial/Control)**	**Duration of treatment**	**Results (statin group compared with the control group)**
Braganza et al., [16]	RCT	Atorvastatin(40mg/day) vs placebo	71	4 weeks	No significant difference
Menzies et al., [21]	RCT	Simvastatin(20mg/day,40mg/day) vs placebo	16	4 weeks	0.86 geometric mean fold decrease in F_E_NO and −0.18 doubling dilution shift in PC10 ↓
Hothersall et al., [22]	RCT	Atorvastatin(40mg/day) vs placebo	54	8 weeks	macrophage count (p=0.029) and sputum fluid leucotriene B4 (p=0.014) ↓
Maneechotesuwan et al., [15]	RCT	Simvastatin(10mg/day vs placebo	47(25/22)	8 weeks	Sputum eosinophil percentages (p=0.02) ↓
Cowan et al., [25]	RCT	Simvastatin(40mg/day) vs placebo	43	4 weeks	Sputum eosinophils (p=0.033) ↓
Al Obaidiet al., [29]	Controlled clinical trial	Simvastatin vs baseline	20	Not mentioned	ECP and CRP ↓

PC10= Concentration of methacholine that reduces FEV_1_ by 10%; F_E_NO= Fractional of exhaled nitric oxide; ECP=Serum eosinophil cationic protein , CRP=C-reactive protein.

↓ The airway and serum inflammation levels were lowering after statins treatment.

### Comparison of symptoms and quality of life

Braganza et al. [16] compared asthmatics treated with atorvastatin (40mg/day) with those treated with placebo in a randomized double-blind parallel group trial. They reported that there was a significant improvement in ACQ score and AQLQ score at 4 weeks in the atorvastatin group without inhaled ICS, which was not maintained at 8 weeks. Another study found that ACQ score was lower in the simvastatin group compared with the placebo group in the absence of steroids [25]. Hothersall et al. [22] showed that there was no significant difference in ACQ score or AQLQ score between atorvastatin and placebo treatment (Table 4).

**Table 4:** Studies which report the comparison of symptoms and quality of life

**Study**	**Study type**	**Group**	**Sample (Trial/Control)**	**Duration of treatment**	**Results (statin group compared with the control group)**
Braganza et al., [16]	RCT	Atorvastatin(40mg/day) vs placebo	71	4 weeks	ACQ and AQLQ (p=0.005) ↑
Hothersall et al., [22]	RCT	Atorvastatin(40mg/day) vs placebo	54	8 weeks	No significant difference
Cowan et al., [25]	RCT	Simvastatin(40mg/day) vs placebo	43	4 weeks	ACQ (p=0.037) ↓

ACQ= Asthma Control Questionnaire; AQLQ= Asthma Quality of Life Questionnaire.

↑ The symptoms and quality of life were improved in statin group compared with the control group, ↓ an opposite result.

### Asthma hospitalization/ED visit, maintenance medications and morbidity

Five studies reported the outcomes of asthma hospitalization/ED visits. Of them, three studies showed that statin exposure was independently associated with a reduction in asthma-related hospitalization/ED visits as indicated by multivariate analysis [13,30,31], whilst the remaining two studies reported contradictory results, one of which reported that statins increased the risk of asthma-related hospitalization/ED visits [18], and the other reported that statins could not reduce this risk, after analysis adjusted for age, sex, baseline severity and comorbidities [17]. An additional two studies showed that statins were associated with a reduction in albuterol or salbutamol use [23,26]. This study also contradicted that of Christiansen et al. who reported that statins did not lead to a decrease the dosage of corticosteroids [17]. Ostroukhova et al. reported that salbutamol was used more frequently in the statin group as compared with the control group [18]. Moskovljevic et al. [28] and Feschenko et al. [14] reported a reduced morbidity score in patients receiving atorvastatin compared with those receiving the placebo. Additionally, a retrospective study showed that the statin groups had more nocturnal awakenings than controls (Table 5) [18].

**Table 5:** Studies which report asthma hospitalization/ED visit, maintenance medications use (steroid or salbutamol) etc

**Study**	**Study type**	**Group**	**Sample (Trial/Control)**	**Duration of treatment**	**Results (statin group compared with the control group)**
Huang et al., [30]	Retrospective study	Statin exposed vs statin unexposed	11808(3965/7843)	4.66 ± 2.32 years	Hospitalization/ED visit (p=0.006) ↓
Ostroukhova et al., [18]	Retrospective study	Statin exposed vs statin unexposed	50(24/26)	2 years	Maintenance medication (p=0.005), nocturnal awakenings(P=0.001), office visits(P=0.003) and albuterol use (p=0.001) ↑
Christiansen et al., [17]	Retrospective study	Statin exposed vs statin unexposed	43158(7783/35375)	Not mentioned	Risk ratios were 1.2 for Hospitalizations/ED visits and 1.17 for oral corticosteroid diepemsing ↑
Stanek et al., [13]	Retrospective study	Statin exposed vs statin unexposed	6574(2103/4471)	1 year	Hospitalization/ED visit (p < 0.001) ↓
Moskovljevic et al., [28]	Controlled clinical trial	Atorvastatin(10mg/day) vs placebo	9	4 weeks	Morbidity ↓
Pagovich et al., [26]	Retrospective study	Atorvastatin, simvastatin vs baseline	70	4 weeks	Albuterol use(p<0.0001) ↓
Fahimi et al., [23]	RCT	Atorvastatin(10mg/day) vs placebo	17	4 weeks	Morbidity (p=0.42) ↓
Feschenko et al., [14]	RCT	Atorvastatin+ICS+Salbutamol vs ICS+salbutamol	31	4 weeks	Night symptoms, cough, dialy symptoms and use of salbutamol (p<0.05) ↓
Lokhandwala et al., [31]	Retrospective study	Statin exposed vs statin unexposed	1437(479/958)	1 year	Hospitalization/ED visit (p=0.0059) ↓

↑ Statin have bad effects on asthma patients, ↓ an opposite result.

## Discussion

A total of 18 studies have been included in this research. Of these, only three studies demonstrated that statins could enhance the lung function in asthmatics, whilst 15 studies failed to support this. There was a tendency that statins were associated with reduced airway inflammation, suggesting that statins may be used as a supplement for anti-inflammatory treatment of asthma at present. The effects of statins were inconsistent in different studies with respect to the symptoms, quality of life, asthma hospitalization/ED visits and maintenance medications. Further large placebo-controlled studies are needed in well-defined subsets of asthmatics patients treated or not treated with corticosteroids before a consensus can be reached.

The clinical status of participants may affect the clinical effectiveness of statins, such as the severity of asthma, age of participants, smoking status and obesity. Feschenko et al. selected 31 severe asthma as the subjects and found that atorvastatin significantly improved clinical and functional outcomes [14], which indicated statins might be effective in severe asthma. However, the results of other included studies, in which the participants were mostly mild to moderate asthmatics did not show a beneficial effect of statins. Further studies on severe asthmatics are needed to confirm this.

A retrospective study found that increasing age was independently associated with an increased risk of hospitalization for asthma patients [30]. This may be due to poor asthma control and medication compliance in elderly patients [32-34] and this may explain the effects of statins.

In addition, some results have shown that ex-smokers and current smokers have a lower decline in FEV_1_ and FVC when taking statins [35,36]. Furthermore, Braganza et al. showed that in smokers with mild to moderate asthma, short term treatment with statins could improve asthma quality of life [16]. Cigarette smoking in asthma is associated with a reduced sensitivity to ICS [3], so statins may act as an effective therapy for asthma patients who have reduced sensitivity to ICS. Finally, studies have demonstrated that obesity was associated with poor asthma control [37,38] and poor response to ICS treatment [39]. Statins are lipid-lowering agents and may be used to relieve the symptoms of obesity-related asthma.

Our analysis of the clinical data reported to date indicates marked differences in study design. Statins inhibit contraction and migration of human airway smooth muscle cells [40]. It was estimated that smooth muscle cells of the mouse aorta divide with a half-life in the range of 300 [41] to 800 [42] days, and this half-life is much longer than the duration of treatment of most clinical studies (4 or 8 weeks). Therefore, if these results are translated to man, the therapeutic effects of statins in asthma may only become apparent after long-term treatment. Long-term studies should be designed to investigate the long- term effects of statins.

There is evidence that the therapeutic effects of individual statins are different. For example, simvastatin and lovastatin are more effective against human smooth muscle cells than atrovastatin in vitro [43]. Furthermore, cerivastatin has the greatest potency in reducing NF-κB-mediated inflammation [44]. Lipophilic statins such as atorvastatin and simvastatin have much greater effects on inflammatory responses in human monocytes in vitro and mice leukocytes in vivo than hydrophilic pravastatin [45]. Thus, new clinical trials should compare the efficacy of different statins since these may have different effects. Animal experiments have confirmed the effective dosage of statins used in animal asthma models, the high-dose being 40mg/kg for simvastatin [11,46] in comparison with 4mg/kg for lovastatin [10]. The maximum recommended dosage of atorvastatin is 80mg/day in man. However, the dosage of statins was below 40mg/day in all eligible studies. In addition, the effect of the small sample size in most of the reported studies must not be ignored.

There are certain limitations of our study. We used a loose selection criteria; for example, abstracts and studies with comparison against baseline only were also selected, because there have not been many studies in this area. In addition, we were unable to conduct a meta-analysis due to limited information, and therefore we could not provide statistically significant evidence to confirm the effectiveness of statins.

In conclusion, statins may reduce airway inflammation in asthmatics, although there is not sufficient evidence to make a conclusion that statins can improve lung function. A sub-population of asthmatics, including smokers and obese individuals who respond poorly to ICS may respond preferentially to statins. The immunomodulating property of statins may shed new light on their promising use in the treatment of asthma. Large sample and multicenter clinical trials in selected specific subpopulations will be needed to investigate the full potential effects of statins in asthma treatment.

## Abbreviations

RCT: randomized controlled trial; ACQ: Asthma Control Questionnaire; AQLQ: Asthma Quality of Life Questionnaire; PC10: Concentration of methacholine that reduces FEV1 by 10%; PEF: Peak Expiratory Flow; FEV1: Forced expiratory volume in one second; FENO: Fraction of exhaled nitric oxide; ACT: Asthma Control Test; PF: Peak flows; ECP: Serum eosinophil cationic protein; CRP: C-reactive protein; FVC: Forced Vital Capacity.

## Competing interests

The authors declare that they have no competing interests.

## Authors’ contributions

All authors read and met ICMJE criteria for authorship. CY and XY designed this study; CY and YT extracted data; CY, LZ, JY.Z, JY.C performed the analysis; CY wrote the first draft of the manuscript and XY, MH, IMA and PJB critically revised the manuscript. All authors read and approved the final manuscript.

## Supplementary Material

Additional file 1 The list of 40 articles that were excluded under the categories of Animal experimentation, Reviews, Trials in vitro and not asthma. Click here for file
