# Supplementary material for: Statins as potential therapeutic drug for asthma?
Source: Respir Res. 2012 Nov 24;13(1):108. doi: 10.1186/1465-9921-13-108 (PMC3545889; doi:10.1186/1465-9921-13-108)
Supplement: Additional file 1 — The list of 40 articles that were excluded under the categories of Animal experimentation, Reviews, Trials in vitro and not asthma. [file 1465-9921-13-108-S1.docx]

**Supplemental table: the list of 40 articles that were excluded under the categories of Animal experimentation, Reviews, Trials in vitro and not asthma.**

|  | Article information ( aothors, title, journal) |
| --- | --- |
| Animal experimentation | Zeki, A. A., L. Franzi, et al. (2009). "Simvastatin inhibits airway hyperreactivity: Implications for the mevalonate pathway and beyond." American Journal of Respiratory and Critical Care Medicine |
|  | Zeki, A. A., J. M. Bratt, et al. (2010). "Simvastatin inhibits goblet cell hyperplasia and lung arginase in a mouse model of allergic asthma: A novel treatment for airway remodeling?" Translational Research |
|  | Yeh, Y. F. and S. L. Huang (2004). "Enhancing effect of dietary cholesterol and inhibitory effect of pravastatin on allergic pulmonary inflammation." Journal of Biomedical Science |
|  | Tschernig, T., W. Baumer, et al. (2010). "Controversial data on simvastatin in asthma: What about the rat model?" J Asthma Allergy |
|  | Rahman, M. N. A. and A. A. M. Abdelmotelb (2011). "Study of Atorvastatin in experimental allergic airway inflammation in mice." International Immunopharmacology |
|  | McKay, A., B. P. Leung, et al. (2004). "A Novel Anti-Inflammatory Role of Simvastatin in a Murine Model of Allergic Asthma." Journal of Immunology |
|  | Liu, M. W., M. X. Su, et al. (2011). "Effect of atorvastatin on airway remodeling and peroxisome proliferator-activated receptor-gamma expression and its mechanism in asthmatic rats." Acta Anatomica Sinica |
|  | Lan, X., S. Yun, et al. (2009). "Simvastatin by inhaled route inhibits airway hyper-responsiveness and inflammation in mice allergic asthma." Annals of Allergy, Asthma and Immunology |
|  | Kagami, S., H. Kanari, et al. (2008). "HMG-CoA reductase inhibitor simvastatin inhibits proinflammatory cytokine production from murine mast cells." International archives of allergy and immunology |
|  | Jacobson, J. R., J. W. Barnard, et al. (2005). "Simvastatin attenuates vascular leak and inflammation in murine inflammatory lung injury." American Journal of Physiology - Lung Cellular and Molecular Physiology |
|  | Imamura, M., K. Okunishi, et al. (2009). "Pravastatin attenuates allergic airway inflammation by suppressing antigen sensitisation, interleukin 17 production and antigen presentation in the lung." Thorax |
|  | Chiba, Y., S. Sato, et al. (2010). "Upregulation of geranylgeranyltransferase I in bronchial smooth muscle of mouse experimental asthma: Its inhibition by lovastatin." Journal of Smooth Muscle Research |
|  | Chiba, Y., S. Sato, et al. (2009). "Lovastatin inhibits antigen-induced airway eosinophilia without affecting the production of inflammatory mediators in mice." Inflamm Res |
|  | Chiba, Y., S. Sato, et al. (2009). "GGTI-2133, An inhibitor of geranylgeranyltransferase, inhibits infiltration of inflammatory cells into airways in mouse experimental asthma." International Journal of Immunopathology and Pharmacology |
|  | Chiba, Y., S. Sato, et al. (2008). "Inhibition of antigen-induced bronchial smooth muscle hyperresponsiveness by lovastatin in mice." Journal of Smooth Muscle Research |
|  | Chiba, Y., J. Arima, et al. (2008). "Lovastatin inhibits bronchial hyperresponsiveness by reducing RhoA signaling in rat allergic asthma." American Journal of Physiology - Lung Cellular and Molecular Physiology |
|  | Chen, Y. J., P. Chen, et al. (2010). "Simvastatin attenuates acrolein-induced mucin production in rats: Involvement of the Ras/extracellular signal-regulated kinase pathway." International Immunopharmacology |
|  | Kim, D. Y., S. Y. Ryu, et al. (2007). "Anti-inflammatory mechanism of simvastatin in mouse allergic asthma model." European Journal of Pharmacology |
|  | Zhu, T., W. Zhang, et al. (2012). "Rosuvastatin attenuates mucus secretion in a murine model of chronic asthma by inhibiting the gamma-aminobutyric acid type A receptor." Chin Med J (Engl) |
|  | Xu, L., X. W. Dong, et al. (2012). "Simvastatin delivery via inhalation attenuates airway inflammation in a murine model of asthma." Int Immunopharmacol |
|  | Aikawa, M., E. Rabkin, et al. (2001). "An HMG-CoA reductase inhibitor, cerivastatin, suppresses growth of macrophages expressing matrix metalloproteinases and tissue factor in vivo and in vitro." Circulation |
| Reviews | Walsh, G. M. (2008). "Defective apoptotic cell clearance in asthma and COPD--a new drug target for statins?" Trends Pharmacol Sci |
|  | Walsh, G. M. (2008). "Statins as emerging treatments for asthma and chronic obstructive pulmonary disease." Expert Review of Respiratory Medicine |
|  | Rubin, B. K. (2009). "Statins for the treatment of asthma: a discovery well, dry hole or just snake oil." Thorax |
|  | Pawlak, J., Z. Zietkowski, et al. (2011). "[Statins and asthma]." Postepy Hig Med Dosw (Online) |
|  | Pawlak, J., Z. Zietkowski, et al. (2009). "Beta-blockers and statins in the context of asthma." Postpy higieny i medycyny do?wiadczalnej (Online) |
|  | Mascitelli, L., F. Pezzetta, et al. (2009). "Statins and cancer in patients with asthma." Thorax |
|  | Feldman, C. (2009). "The role of statins in respiratory diseases." Clinical Pulmonary Medicine |
|  | Zeki, A. A., N. J. Kenyon, et al. (2011). "Statin drugs, metabolic pathways, and asthma: A therapeutic opportunity needing further research." Drug Metabolism Letters |
|  | Van Asperen, P. (2011). "What's new in the management of asthma in children?" Medicine Today |
|  | Sharma, P. and A. J. Halayko (2009). "Emerging molecular targets for the treatment of asthma." Indian Journal of Biochemistry and Biophysics |
|  | Royce, S. G. and M. L. K. Tang (2009). "The effects of current therapies on airway remodeling in asthma and new possibilities for treatment and prevention." Current Molecular Pharmacology |
| Trials in vitro | Takeda, N., M. Kondo, et al. (2006). "Role of RhoA inactivation in reduced cell proliferation of human airway smooth muscle by simvastatin." American Journal of Respiratory Cell and Molecular Biology |
|  | Samson, K. T. R., K. Minoguchi, et al. (2006). "Inhibitory effects of fluvastatin on cytokine and chemokine production by peripheral blood mononuclear cells in patients with allergic asthma." Clinical and Experimental Allergy |
|  | Robinson, A. J., D. Kashanin, et al. (2009). "Fluvastatin and lovastatin inhibit granulocyte macrophage-colony stimulating factor-stimulated human eosinophil adhesion to inter-cellular adhesion molecule-1 under flow conditions." Clinical and Experimental Allergy |
|  | Luo, F. M., C. T. Liu, et al. (2005). "Simvastatin induces eosinophil apoptosis in vitro." Zhonghua Jiehe he Huxi Zazhi |
|  | Krimmer, D. I., J. K. Burgess, et al. (2011). "Simvastatin attenuates cigarette smoke extract induced production of extracellular matrix in vitro." Respirology |
| Not asthma | Wang, W., W. Le, et al. (2011). "Inhibition of inflammatory mediators: role of statins in airway inflammation." Otolaryngol Head Neck Surg |
|  | Keddissi, J. I., W. G. Younis, et al. (2007). "The use of statins and lung function in current and former smokers." Chest |
|  | Alexeeff, S. E., A. A. Litonjua, et al. (2007). "Statin use reduces decline in lung function: VA Normative Aging Study." Am J Respir Crit Care Med |
